# Using fNIRS to Identify Transparency- and Reliability-Sensitive Markers of Trust Across Multiple Timescales in Collaborative Human-Human-Agent Triads

**DOI:** 10.3389/fnrgo.2022.838625

**Published:** 2022-04-07

**Authors:** Lucca Eloy, Emily J. Doherty, Cara A. Spencer, Philip Bobko, Leanne Hirshfield

**Affiliations:** ^1^Institute of Cognitive Science, University of Colorado Boulder, Boulder, CO, United States; ^2^Department of Management, Gettysburg College, Gettysburg, PA, United States

**Keywords:** fNIRS (functional near-infrared spectroscopy), human-agent teaming, human-agent trust, agent transparency, trust prediction, neural correlates of trust, agent reliability, mental demand

## Abstract

Intelligent agents are rapidly evolving from assistants into teammates as they perform increasingly complex tasks. Successful human-agent teams leverage the computational power and sensory capabilities of automated agents while keeping the human operator's expectation consistent with the agent's ability. This helps prevent over-reliance on and under-utilization of the agent to optimize its effectiveness. Research at the intersection of human-computer interaction, social psychology, and neuroergonomics has identified trust as a governing factor of human-agent interactions that can be modulated to maintain an appropriate expectation. To achieve this calibration, trust can be monitored continuously and unobtrusively using neurophysiological sensors. While prior studies have demonstrated the potential of functional near-infrared spectroscopy (fNIRS), a lightweight neuroimaging technology, in the prediction of social, cognitive, and affective states, few have successfully used it to measure complex social constructs like trust in artificial agents. Even fewer studies have examined the dynamics of hybrid teams of more than 1 human or 1 agent. We address this gap by developing a highly collaborative task that requires knowledge sharing within teams of 2 humans and 1 agent. Using brain data obtained with fNIRS sensors, we aim to identify brain regions sensitive to changes in agent behavior on a long- and short-term scale. We manipulated agent reliability and transparency while measuring trust, mental demand, team processes, and affect. Transparency and reliability levels are found to significantly affect trust in the agent, while transparency explanations do not impact mental demand. Reducing agent communication is shown to disrupt interpersonal trust and team cohesion, suggesting similar dynamics as human-human teams. Contrasts of General Linear Model analyses identify dorsal medial prefrontal cortex activation specific to assessing the agent's transparency explanations and characterize increases in mental demand as signaled by dorsal lateral prefrontal cortex and frontopolar activation. Short scale event-level data is analyzed to show that predicting whether an individual will trust the agent, with data from 15 s before their decision, is feasible with fNIRS data. Discussing our results, we identify targets and directions for future neuroergonomics research as a step toward building an intelligent trust-modulation system to optimize human-agent collaborations in real time.

## Introduction

### Trust and Human-Agent Teams

With rapid advances in the human-agent teaming (HAT) research domain, intelligent agent systems are expected to improve the quality of human-agent collaborations in almost all domains (e.g., military, business, medical, educational, etc.). In a recent white paper on HATs in the military, DeCostanza et al. (2018, p. 2) state “The concept of technology being a tool for humans will be superseded by technology as mentored actors in the environment, teammates with unique non-human skills, and technology that augments fundamental human capabilities.” Following this philosophy, we adopt a multi-person HAT framework to investigate the neuroergonomics of hybrid human-agent teams in a naturalistic, open-ended task.

As human and AI agents increasingly become interdependent and interactive *teammates* (e.g., Chiou and Lee, 2021), successful HAT systems must leverage the computational power and sensory capabilities of computer agents to support the human operator(s)' decision making, allowing them to offload tasks onto the agent when appropriate to increase team efficiency and performance. Trust, generally defined as a willingness to accept vulnerability based on positive expectations of others (Rousseau et al., 1998), drives human decision-making when offloading collaborative tasks (Lee and See, 2004; Mouloua and Hancock, 2019). Thus, it has become the target variable of much HAT research seeking to dynamically tune an agent's characteristics and behavior to encourage proper trust calibration. Trust *calibration* refers to a human operator maintaining an appropriate level of trust to think systematically and rationally about the capabilities of the agent (Lee and See, 2004). Other factors like the operator's own level of workload and ability must also be considered when calibrating trust. Indeed, “overtrust” can result in automation-induced complacency which causes overreliance on an assistive agent in situations the agent is not equipped to handle (Parasuraman et al., 1993). On the other hand, “undertrust” induces an operator to reject useful assistance, thus failing to take advantage of the agent's computational potential and increasing their own task load. Consequently, trust must be dynamically tuned to accurately reflect the agent's capability at a given task.

#### Influencing Trust in Agents: Transparency and Reliability

In extending trust research and theory to adaptive systems, several reviews (Lee and See, 2004; Madhavan and Wiegmann, 2007; Parasuraman et al., 2008; Hoff and Bashir, 2015; Glikson and Woolley, 2020) have identified factors that influence human trust in an agent, largely based on Lee and See's (Lee and See, 2004) “purpose, process, & performance” model of trust in automation. The more recent of these reviews (Hoff and Bashir, 2015; Glikson and Woolley, 2020), focuses individually on trust in robotic, virtual, and embedded AI systems, emphasizing (i) transparency and (ii) reliability as important trust-building factors. Transparency refers to the extent to which an agent's underlying operation and decision-making is made apparent to the operator, often through an explanation or rationale (Hoff and Bashir, 2015). Reliability, or the level to which an automation's behavior is accurate and consistent, will always be subject to “real-world” factors outside of experimental contexts; changes in reliability are an inevitable consequence of imperfect AI. A recent study (Hussein et al., 2020) examined the specific effects of transparency and reliability on trust, finding that trust built primarily from reliability increased participant reliance on the agent (in this case, a simulated swarm of drones) compared to trust built from transparency. Transparency-based trust, however, increased the number of correct rejections of the agent's recommendation (and thereby human performance on the task), whereas reliability-based trust led to fewer rejections overall (potentially leading to over-trust or complacency). This finding aligns partially with other studies concluding that reliability without transparency can lead to human overreliance on an agent during a task (Chancey et al., 2015; Wang et al., 2016; Hussein et al., 2020). Indeed, the goal of transparency is to appropriately calibrate a human operator's trust and reliance on the agent depending on the agent's level of certainty in the task (see also Kunze et al., 2019; Bhaskara et al., 2020; Hussein et al., 2020 for a review). This calibration— e.g., decreasing trust in situations of low certainty and increasing trust in situations of high certainty— makes human-agent teams more effective. For example (Kunze et al., 2019), used a simulated heartbeat to display an autonomous driving system's level of certainty which, compared to the control group, resulted in drivers taking over significantly faster before a collision event. We note here that there are a number of ways for an agent to leverage its internal algorithms and output a confidence level for each recommendation or action given by the agent (Hagras, 2018; Zhang et al., 2020).

Considering the specific effects of transparency, if the actual transparency communication is complex to interpret (e.g., comprehensive visualizations) prior evidence intuitively suggests an increase in workload due to a higher demand in information processing (Helldin, 2014; Kunze et al., 2019; Akash et al., 2020). However, if the transparency manipulation is quick and relatively easy to interpret, a measurable increase in workload may be avoided. This is thoroughly discussed in recent work by Miller on the lifecycle of trust in HATs, which suggests that agent transparency while teams are “in action” should be designed to eliminate unnecessary workload and allow teams to focus their efforts on the task at hand (Miller notes that other times in a team's lifecycle, such as in a post-action review, are better times to build trust and show agent transparency in more complex, information-rich ways) (Miller, 2021). We outline our investigation of these transparency effects in hypothesis 1 below.

### Measuring Trust With Neurophysiological Sensors

To study and develop trust-calibration systems, most efforts rely on periodic surveys and behavioral measures of latent trust such as task compliance (willingness to accept an agent's recommendation). While this is a necessary characteristic of experimental paradigms, further applied development of human-agent teaming systems in naturalistic settings requires a constant, undisruptive measure of human trust in the agent independent of the task itself. One challenging but promising direction is the use of neurophysiological sensor data and machine learning to predict levels of trust and other relevant latent variables. Although fMRI remains the standard for precise measurements of the functioning human brain, it is not feasible for real-time measurement during human-agent teaming due to the cost and physical restrictions (participants must be inside the scanner). Less invasive technologies have become an attractive alternative as their lightweight sensors, usually consisting of a headcap with probes, allow studies in a much wider variety of environments.

In particular, we note that functional near-infrared spectroscopy (fNIRS) holds great potential for measurement of trust and other cognitive, social, and affective states in ecologically valid contexts (Curtin and Ayaz, 2018; Hirshfield et al., 2019). While the high temporal resolution of EEG allows for precise measurement of neural events in a non-invasive package, fNIRS is a more spatially robust technology that shows future promise with rapidly advancing usage and analysis techniques. In a recent review, Curtin and Ayaz identify fNIRS as one of the most promising technologies for applications of continuous brain monitoring, highlighting the technology's ecological advantages and suitability for applied neuroergonomics (Curtin and Ayaz, 2018). fNIRS uses infrared light to measure hemodynamic responses that occur in the brain following neuronal activation— specifically, Δoxyhemoglobin (HbO) and Δdeoxyhemoglobin (HbR)—and thus boasts higher spatial resolution than EEG and temporal resolution comparable to fMRI. Furthermore, fNIRS devices are easily configurable, do not require any gel or special preparation, and can function wirelessly allowing for virtually no movement restriction while in use.

#### Neurophysiological Markers of Trust: Prior Evidence

Since fNIRS remains in its nascent stage when compared EEG and fMRI, few studies have used the device to measure complex social constructs, such as trust and suspicion, in the brain. fMRI, on the other hand, has been used to measure the neural correlates of trust and distrust in countless studies; this body of literature is often used by fNIRS researchers as a roadmap in this domain (Krueger et al., 2007; Dimoka, 2010; Bhatt et al., 2012; Fett et al., 2014). Results from these studies can be difficult to collate due to differences in experimental tasks and in the operationalization of trust. See Hirshfield et al. (2019) for a summary of the primary functional brain regions implicated in trust and social reasoning across a wide swath of trust fMRI studies. More specifically, brain regions including the frontopolar area (FPA), medial prefrontal cortex (MPFC), dorsolateral prefrontal cortex (DLPFC), and the bilateral temporoparietal junction (TPJ) have often been linked to trust, decision making, and interpersonal reasoning (Watabe et al., 2011; Aimone et al., 2014; Fett et al., 2014; Mahy et al., 2014; Pushkarskaya et al., 2015; Filkowski et al., 2016; Nozawa et al., 2016; Hirshfield et al., 2019; Salazar et al., 2021).

Activity in MPFC and TPJ has been specifically linked to Theory of Mind reasoning, a paradigm that involves perceiving the intentions of others in relation to one's own thoughts and actions (Sebastian et al., 2012; Mahy et al., 2014). *Dorsal* MPFC (DMPFC) in particular has been linked to reasoning and making social judgements about others (vs. oneself) during decision-making (Mitchell et al., 2005), a process crucial to evaluating one's intent when deciding to trust (recall Mayer et al.'s ability, benevolence, and integrity model of trust). A large meta-analysis of neuroimaging studies confirmed evidence for DMPFC's specificity toward reasoning and judgement about others, also implicating TPJ in similar social reasoning and decision-making processes (Denny et al., 2012). Tang et al. (2016) used fNIRS to measure brain activation of two individuals during a face-to-face economic exchange and found the right TPJ to be more active during increased intentionality and collaborative interactions (Tang et al., 2016).

DLPFC has often been implicated in executive processes, such as task supervision and affect regulation, and as a marker of mental demand. Bunce et al. used fNIRS to investigate hemodynamic responses in the DLPFC in relation to workload and development of expertise, finding that DLPFC activation increased with mental demand only to the extent that participants are able to perform in the task (Bunce et al., 2011). Subsequent studies have consistently validated and improved implementations of continuous workload monitoring using optical neuroimaging (Ayaz et al., 2012; Durantin et al., 2014; Liu et al., 2017; Curtin and Ayaz, 2018). More recently, McKendrick et al. used a robust exploratory modeling procedure to specify a nonlinear (cubic polynomial) relationship between working memory load and HbO changes in left DLPFC as measured by fNIRS (McKendrick and Harwood, 2019). Their findings characterize this complex relationship, suggesting that a change in workload states (as opposed to just the state itself) can disrupt cognitive processes and performance.

In line with the findings above, Hirshfield et al. (2019) conducted a computer-mediated interpersonal survival task in which participants made survival decisions based on information from a confederate. They used a General Linear Model analysis on fNIRS recordings and found the frontopolar area (FPA), TPJ, and DLPFC significantly more activated during a suspicion condition than during a trustworthiness condition (Hirshfield et al., 2019). Furthermore, those authors trained an LSTM classifier to predict suspicion with an average of 76% accuracy, using leave-one-participant-out cross validation, highlighting the predictive power of these regions-of-interest (ROIs) for future applications of adaptive trust-modulation systems. These regions reside in the outer cortex, making them prime targets for fNIRS research as the NIRS signal cannot penetrate deeper into the brain. We therefore selected fNIRS optode locations to maximize coverage of these areas in the present study; **Figure 3** depicts this montage of NIRS source-detector pairs.

### Objectives and Hypotheses

Few studies have attempted to classify trust in automated agents using neurophysiological signals in naturalistic settings. Those that do exist employ very controlled but hyper-specific tasks that reduce the participants' potential decisions and freedom in approaching the task (Akash et al., 2018; Wang et al., 2018; Gupta et al., 2019). While promising, these studies are largely unable to draw any clear functional neuroergonomic conclusions from their results, highlighting the need for more robust, ecologically valid, and generalizable studies of neurophysiological modeling of human-agent trust. Furthermore, much prior work is limited by its use of HATs with only one human operator. To the authors' knowledge at the time of writing, no studies involving neurophysiological sensing of trust in multi-human HATs exist. However, this existing literature provides a clear direction and set of unanswered questions to build upon.

The present study seeks to begin addressing this gap with an open-ended, highly collaborative, and asymmetrical triadic HAT task. We aim to investigate the social, cognitive, and affective hemodynamics of trust in teams of two human participants and one assistive agent. To accomplish this goal, we developed a geospatial allocation task in which human and agent teammates are tasked with exploring real historical crime data to decide on placements of crime-prevention resources in a metropolitan area. Drawing from previous research outlined above, we manipulate transparency and reliability of the agent to assess the effects on trust, reliance, mental demand, affect, and team dynamics. We further investigate patterns of activation in brain ROIs to identify functional correlates of trust that show promise for future research on detection and calibration of human-agent trust.

Although the research described above can be complicated to synthesize, at times using different constructs and operationalizations of trust, and a myriad of experimental designs ranging in task complexity, some general trends to emerge. We examine these trends to formulate and present our research goals and hypotheses:

Hypothesis 1: When easy-to-interpret transparency yields calibrated trust, we expect to see high transparency yield lower mental effort as compared to low transparency—that is, the decision to trust or distrust an agent action is made with relative ease when transparency is high. This is opposed to the case of low transparency, where the operator must devote mental energy while in a state of suspended judgement, not yet trusting or distrusting the agent's recommendation. In line with prior work (Chen et al., 2011; Hu et al., 2016; Hirshfield et al., 2019) we expect to see an increase in mental demand (accompanied by increased arousal) following this state of suspended judgement, until the participant decides to accept or reject an agent recommendation. We posit that behavioral, self-report, and neuroimaging measurements, assessed *via* fNIRS, will triangulate to the expected responses above between transparency conditions. Specifically, we expect response time and mental demand self-report scores to increase and agent trust scores to decrease in low transparency conditions as participants must make a credibility assessment for each of the agent's recommendations.

Hypothesis 2: Regarding agent reliability, many articles note (as is logically expected) that increased automation reliability is associated with increased levels of trust and reliance (e.g., Parasuraman and Riley, 1997; Lee and See, 2004; Wright et al., 2019). Subsequently, high reliability can lead to a decrease in mental demand as participants are more inclined to offload effort onto the agent. We expect agent trust survey scores and our behavioral measure of reliance (number of agent recommendations accepted) to be lower when reliability is low. Following the effects outlined above, we also expect an increase in mental demand reflected in survey scores and response time.

Hypothesis 3: Toward our eventual goal of real-time trust measurement and adaptation, we seek to evaluate the predictive power of neuroimaging features by focusing on short-term moments of trust. Based on prior fNIRS research, we expect to see significant increases in activation across brain ROIs leading up to non-trusting decisions. We postulate that activation in DLPFC, FPA, and TPJ will be identified as useful moment-level predictors of trust in the agent.

## Methods

### Data Collection

#### Participants

Participants were *N* = 38 students (average age 21, 53% male) from a large public university. Students completed the task in teams of 2 participants and 1 agent, for a total of 19 teams/sessions. Participants were compensated monetarily for their time ($15/h) along with a variable cash bonus based on task score. Recruitment and experimental procedures were approved by the university's Institutional Review Boards. Participants completed informed consent forms upon arriving at the lab.

#### Testbed Environment

**Figure 1:**
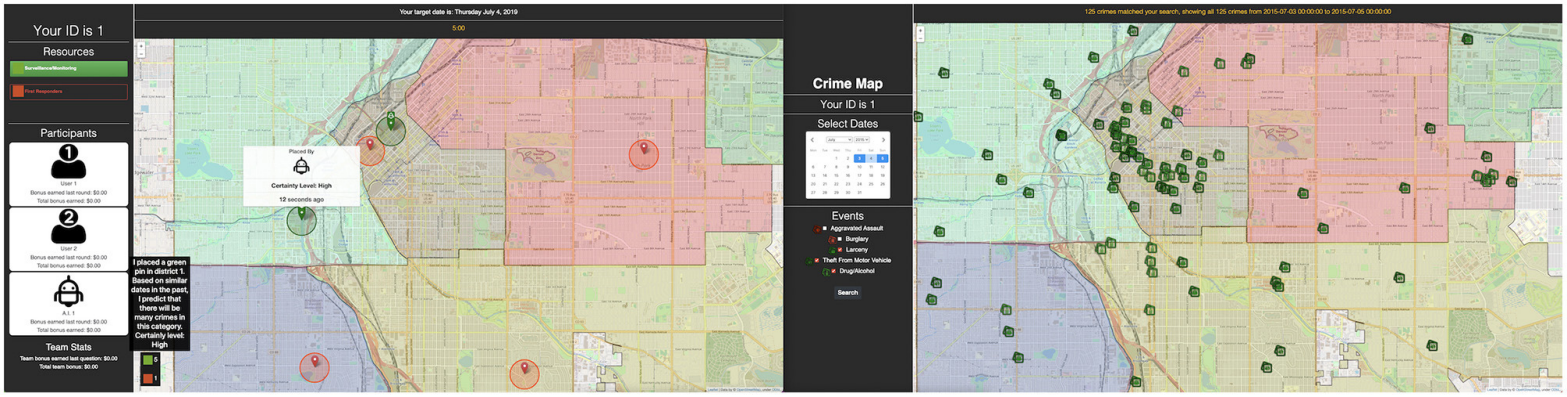
Screenshot of each participant's dual monitor screen. The right side monitor has each participant's map for searching crime data. The left side monitor is a shared map where each teammate (human and AI) dynamically places, moves, or deletes pins (their own or teammates' pins).

The experimental task was conducted using the Computer-Human Allocation of Resources Testbed (CHART, depicted in Figure 1), developed by the researchers for the present study. CHART allows a human-agent team to collaborate on a crime mapping task, in which participants search through past spatiotemporal crime data in Denver, CO and allocate a limited number of crime prevention resources throughout the city. Each team's shared goal is to “catch” as many crimes as possible through resource allocation. The interface consists of two displays, shown in Figure 1.

The right display is an interactive crime map that allows participants to select a range of past dates and category(s) of crimes (e.g., traffic incidents, assault, theft) to overlay on a map of Denver. Each user can explore their crime map independently. All crime data included is publicly available via the Denver Police Department. The left display consists of a shared map (i.e., changes on this map are reflected across all teammates' screens) on which participants place their resources (represented as pins), as well as a sidebar detailing the team's current cash bonus as well as each individual's contribution, based on their performance on prior rounds. Participants allocate resources by placing pins on the shared map, as well as moving and deleting pins.

Two kinds of resources, differentiated by pin color, are available and each correspond to a unique set of crimes. Green pins, labeled as “surveillance/monitoring” resources, are able to prevent non-violent crimes; red pins, or “first responder” resources, address violent crimes. To simulate expertise in a “real-world” collaboration, each team member is given access to different categories of historical crime data and must therefore share knowledge with other teammates to perform well. Table 1 contains a complete list of crimes in each category and which user can access them. The crime layers available to each user are selected to ensure that both participants have roughly equivalent amounts of information (i.e., a similar number of total crime events). Participants have an allotment of 6 green pins and 6 red pins (shared between the two) to place during each round. The rest of the pins are placed by the “AI” agent (detailed below) and can be moved by either participant.

**Table 1:** Type of crime, associated crime layers in the testbed per user, and corresponding color of resource pin.

**Crime category**	**Layers**	**Color of resource pin**
Violent crime	• User 1: *aggravated assault, burglary, larceny*	Red
	• User 2: *murder, robbery, arson*	
Non-violent crime	• User 1: *theft from motor vehicle, drug/alcohol*	Green
	• User 2: *traffic accident, auto theft*	

*Participants could filter the crime map by selecting which categories of crime to display*.

#### AI Agent and Manipulations

CHART also allows for the use of a pre-programmed (wizard-of-oz-style) “AI” agent, which was used in the present study to simulate a multiparty human-agent team. Throughout each round, the agent places 6 green pins and 6 red pins in alternating order. Participants are told that the agent is one of their team members and its pin placements *do* contribute to their cumulative cash bonus. When the agent places a pin, an alert tone is emitted through the computer speakers, and a textbox appears in which the agent announces its placement and, depending on the experimental (transparency) condition, provides a justification. Pins placed by the agent can be moved by either participant, but not deleted. In this study, we manipulated the reliability (high/low) and transparency (high/low) of the agent teammate.

**Figure 2:**
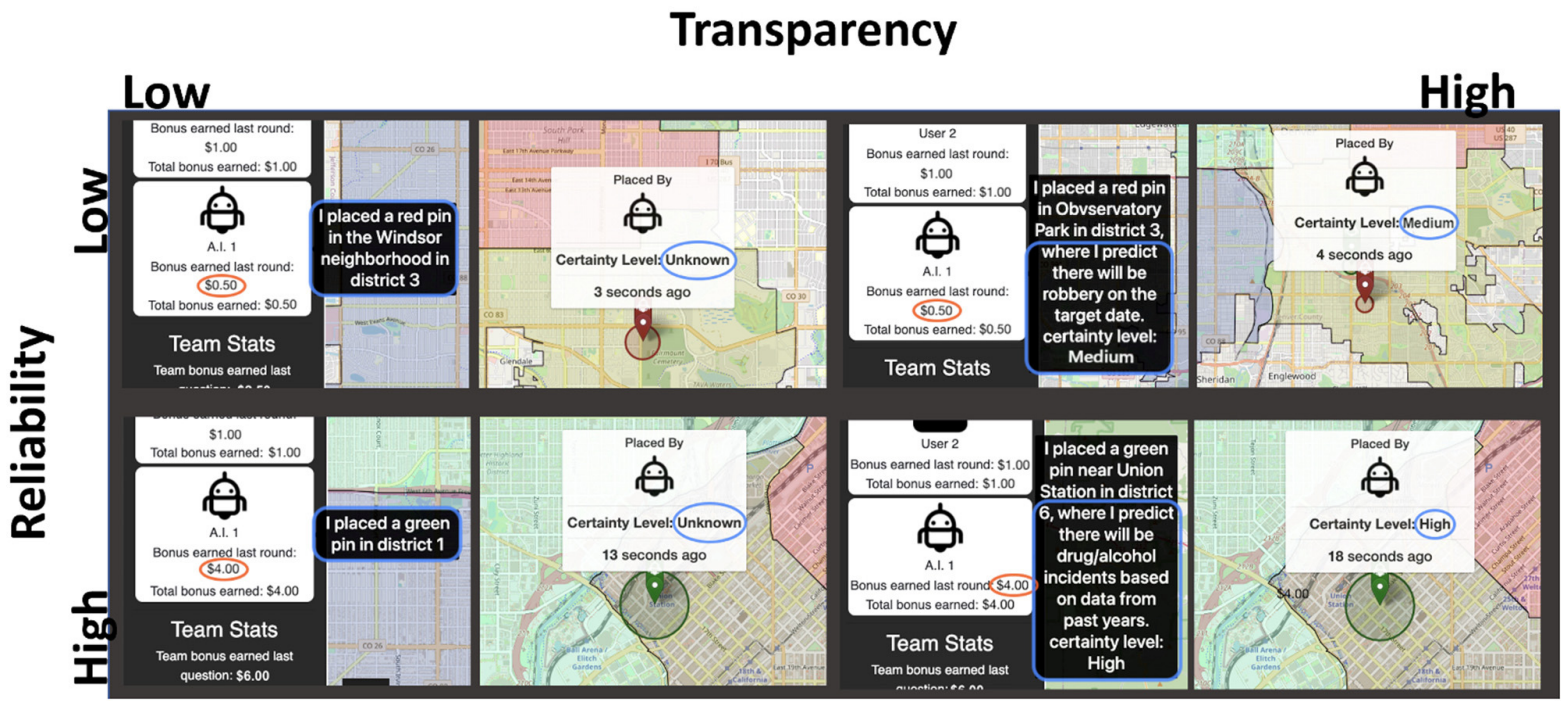
Example of transparency (high/low) and reliability (high/low) manipulations in experiment.

Given that transparency also includes an indication of certainty (see Bhaskara et al., 2020's review), participants could also view the agent's certainty by hovering over the pin with their mouse. In high transparency conditions, a pop-up text message indicated that certainty levels were either “high or “medium.” In low transparency conditions, certainty levels were always listed as “unknown.” Since machine learning algorithms have several ways for a model to provide information about its level of certainty for a given prediction, this information is deemed a useful way for agents to provide information about their confidence in a given prediction (Zhang et al., 2020). In high reliability conditions the agent's internal representation for each of 12 pin placements was “high” for 9 days and “medium” for 3 days. Conversely, certainty levels for low reliability conditions were “medium” on 9 days and “high” on 3 days. Participants are told in their instructions that “some dates are easier than others for the AI to find patterns and trends (for example, sometimes there's not enough past crime data to make a clear prediction).” Figure 2 shows an example of the AI's pin placements under each condition.

#### Experimental Procedure

Upon arriving at the lab, participants completed informed consent forms and were then led to separate rooms each with a desk and desktop computer on which they completed the task. They were first instructed to complete a pre-survey on the computer, after which the experimenter opened a slideshow presentation containing instructions on how to complete the task (with text descriptions and short animations of possible actions). Participants were instructed to read through the slideshow at their own pace. After reviewing the instructions each participant independently completed a CHART training session consisting of two shortened (2 min) rounds, during which the agent places only 2 pins, in order to learn how to use the CHART interface.

Once both participants completed their training sessions, they were each outfitted with a NIRx NIRSport2 headcap, a Shimmer GSR+ sensor, and calibrated a Tobii 4c mounted eye-tracker. Next, a Zoom call between the two participants was initiated to facilitate communication. Participants were then directed to click “I'm Ready” on the testbed when they were ready to begin, and they were left alone in their respective rooms. The first round began once both participants select “I'm Ready” and ended when the 6-min timer reached 0. After each round, a score table presented each individual's and the team's total bonus earned. Participants then selected a “start survey” button which opened a Qualtrics window with a short post-round survey (average survey completion time = 106 s). Once both participants completed their surveys, they were able to select “I'm Ready” once again to begin the next round. This procedure was repeated until the conclusion of the 8th round, after which a final post-round survey was completed and the experimenters removed the sensors and paid participants for their time plus bonus earned.

### Measures

Self-report surveys, administered after each experimental round, were selected as dependent measures of emergent individual and team states, including affect, trust, mental demand, and team processes. Due to the length of the study, we chose only the items with the highest factor loadings to be able to assess constructs of interest while minimizing time spent on surveys. Behavioral measures were extracted after data collection by a script using CHART's comprehensive event logs. Timestamps were used to sync neurophysiological time series data with experimental rounds and events.

#### Surveys

##### Trust in the Agent

Trust was assessed using four items from Merritt's (2011) six-item scale of trust in automation. These items were selected to be straightforward and adaptable to minimize surveys' disruption of the task. Example items include “I trust the AI agent on my team” and “I have confidence in the advice given by the AI agent” (Merritt, 2011). The final “trust in the agent” score was computed as an average of the four items. Cronbach's alpha for these items was α = 0.88.

##### Trust in Teammate

To keep post-round surveys concise, trust in teammates was assessed simultaneously with trust in the agent by modifying items from the above scale to refer to the human teammate. Example items therefore read “I trust the other person on my team” and “I have confidence in the advice given by the other person on my team.” Responses were averaged across the four items. Cronbach's alpha for these items was α = 0.91.

##### Team Processes

We assessed team processes under the framework proposed by Marks et al. (2001) by selecting the top factor loading items from each section of Mathieu's team for brevity. We included items to measure coordination, conflict management, goal monitoring (taken from “Monitoring” items), strategy formulation, and cohesion (from “Affect Management” items) (Mathieu et al., 2020). Responses were averaged across the five items. Cronbach's alpha for these items was α = 0.93.

##### Mental Demand

Mental demand was assessed using the corresponding item from the NASA-TLX using an on-screen slider. Scale scores ranged from 0 (“very low”) to 20 (“very high”). Previous work has shown this subscale to be most closely linked to task demand (McKendrick and Cherry, 2018).

##### Perceived Performance

Similarly, perceived performance was assessed using the performance item from the NASA-TLX using an on-screen slider. Scale scores ranged from 0 (“very low”) to 20 (“very high”).

##### Valence and Arousal

Russell (1980) provides a classic, circumplex model of affective states which is defined by two coordinate dimensions of valence and arousal (Russell, 1980). We used measurement scales that were based on this work and found in more recent studies in information technology (Hussain et al., 2011; Eloy et al., 2019). Using Likert scales, participants rated their emotional valence on a scale from 1 (“very negative”) to 5 (“very positive”); they also rated their emotional arousal on a scale from 1 (“very sleepy”) to 5 (“very active”).

#### Behavioral Measures

##### Reliance

Reliance on the agent was measured behaviorally— by counting the number of pins placed by the agent that each individual subsequently moved during a round. In other words, we measured how often individuals rejected the agent's recommendation. Given that the agent was programmed to place 12 pins in each round, we subtracted the number of pin movements from 12, so that higher scores were associated with higher reliance.

##### Response Time

Response time was chosen as a measurable correlate of mental demand (Gvozdenko and Chambers, 2007; Akash et al., 2020) by calculating the average time lapse (for each individual) between the agent placing a pin and the individual moving the pin. Only cases where the pin was moved were taken into consideration, which resulted in the few individuals who never moved a pin missing a reaction time value.

#### Neurophysiological Sensors

**Figure 3:**
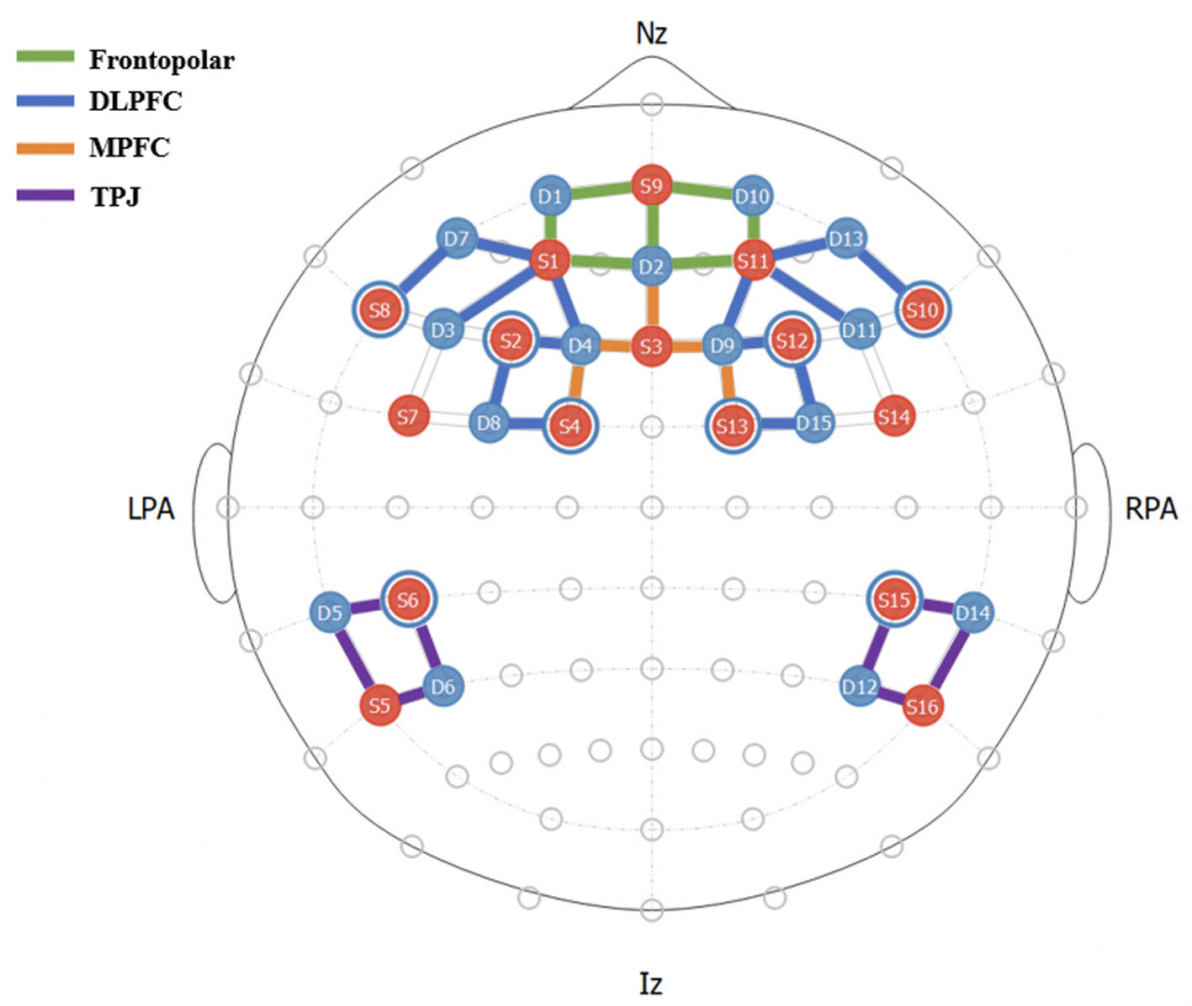
fNIRS Montage and ROI Mappings. Each channel consists of a source-detector (S#-D#) pair, represented by colored lines.

Data were recorded on 38 participants using 2 NirSport 16x16 machines (NIRx, Berlin, Germany) with a sampling rate of 5.0863 Hz. The montage of 42 channels was divided into 4 regions-of-interest (ROIs): frontopolar, DLPFC, MPFC, and TPJ regions using fOLD Toolbox (Morais et al., 2018). Figure 3 shows placement of channels and their assigned ROIs.

## Results

### Experimental Results

First, a manipulation check was performed by regressing several outcome variables on transparency and reliability to see if the manipulations had direct effects on human teammates' social, cognitive, and affective states. We used linear mixed-effects modeling using the R package lme4 (Bates et al., 2015) to account for the repeated measures (multiple rounds) and nested (individual participants within teams) structure of the data. In addition to transparency and reliability conditions, round number (recall there were 8 rounds for each team) was included as a covariate to control for effects of time. Nested random effects took the form (1|team/participant) as similarities are expected between members of the same team and measures from the same individual. Table 2 presents results across dependent measures.

**Table 2:** Results of mixed-effects modeling of transparency and reliability effects on response variables.

**Response**			**Agent**	**Mean response**	**Teammate**	**Team**	**Perceived**	**Mental**		
**variables**		**Agent trust**	**reliance**	**time**	**trust**	**processes**	**performance**	**demand**	**Valence**	**Arousal**
Transparency [Low]	*Estimate(std err)*	−0.30 (0.08)	−0.34 (0.20)	−3.74 (6.14)	−0.13 (0.05)	−0.15 (0.05)	−0.33 (0.36)	0.30 (0.20)	−0.15 (0.08)	0.08 (0.08)
	*p*	**<0.001**	0.092	0.543	**0.012**	**0.001**	0.367	0.128	0.072	0.328
Reliability [Low]	*Estimate (std err)*	−0.31 (0.08)	−0.32 (0.20)	13.88 (6.13)	−0.04 (0.05)	0.01 (0.05)	−1.07 (0.36)	0.37 (0.20)	−0.29 (0.08)	−0.13 (0.08)
	*p*	**<0.001**	0.120	**0.024**	0.411	0.797	**0.005**	0.058	**0.001**	0.123
Round	*Estimate (std err)*	−0.01 (0.02)	−0.11 (0.04)	−1.92 (1.39)	0.03 (0.01)	0.05 (0.01)	0.22 (0.08)	−0.05 (0.04)	0.01 (0.02)	−0.08 (0.02)
	*p*	0.682	**0.012**	0.166	**0.002**	**<0.001**	**0.005**	0.205	0.653	**<0.001**

*“Low” condition is given as a reference level for transparency and reliability. Bolded values indicate p < 0.05*.

#### Do Agent Transparency and Reliability Manipulations Affect Trust and Outcome Measures?

##### Transparency

Following H1, trust in the agent was lower (*B* = −0.30*, p* < 0.01) in low transparency compared to high transparency conditions. However, no effect of transparency was seen on mental demand (*B* = −0.37*, p* < 0.128).

Low transparency also corresponded to decreased teammate trust (*B* = −0.13*, p* = 0.01) and team processes (*B* = −0.15*, p* < 0.01). Valence and arousal were not affected by transparency as we expected. These results suggest that omission of agent communication disrupted the cohesion of both human-human and human-agent dynamics.

##### Reliability

Average response time in seconds (*B* = 13.88*, p* = 0.02) significantly increased and mental demand scores (*B* = 0.09*, p* = 0.06) showed a marginal effect when agent reliability was low, while perceived performance decreased (*B* = −1.07*, p* < 0.01). Results indicate that lower agent performance also increased perceived difficulty and participant mental demand, but did not significantly influence teammate trust (*B* = −0.04*, p* = 0.41) or team processes (*B* = 0.01*, p* = 0.80). Lastly, we found lower agent reliability negatively proportional to emotional valence (*B* = −0.29*, p* < 0.01) but not arousal (*B* = −0.13*, p* = 0.12). Reliance on the AI (measured by the number of pins accepted), however, did not vary significantly between conditions as we predicted in H2 (*B* = −0.32*, p* = 0.12).

Analysis of interaction effects between transparency and reliability manipulations did not return any significant results across outcome measures.

### fNIRS Results

To examine the neural markers of trust-relevant agent manipulations, we first analyzed fNIRS data for each entire round to match the granularity of our experimental manipulations and the survey measures administered at the end of each round. Our goal was to identify specific differences in brain activity to better understand the neural processes involved in human-agent teaming under different conditions. We then investigate the potential for brain activation features to states of trust under varying agent behaviors.

fNIRS data were processed in NirsLAB (V2019.04, NIRx). All rounds were truncated to 1896 timeframes (373s) to reduce variability among participants as performed by other researchers (Hawkins et al., 2018). Each time series was truncated to only include 5 s before the first round and 32 s following the last round to include the full hemodynamic response. Data quality was checked for coefficient of variation (CV), a signal-to-noise ratio measure, and channels with higher CV were visually examined (visual inspection clearly reveals whether a single motion artifact is skewing CV or the optode did not have adequate contact with the scalp); if too much noise was detected, they were excluded from analysis. CV is a widely used procedure for the filtering of raw light intensity measurements by fNIRS (Schmitz et al., 2005; Schneider et al., 2011). We chose a CV cutoff of 15% and therefore channels with a CV equal or >15% were more closely inspected, as used by other researchers (Piper et al., 2014; Pfeifer et al., 2018). Three participants were excluded from analysis: two participants had missing data due crashing of the NirSport and one other participant had all 42 channels marked as “bad” with CV equal or higher than 15%. We followed pre-processing guidelines outlined by leaders in the fNIRS community in their 2021 publication (Yücel et al., 2021). A pre-whitening autoregressive model-based algorithm (Barker et al., 2013) was applied to the data to correct for motion and serially correlated errors, as recommended by Yücel et al. Prewhitening the signal before analysis removes confounding signals like motion artifacts and physiological oscillations (such as the Mayer wave). This specific prewhitening algorithm requires that no prior traditional filtering is applied (low-pass, high-pass, bandpass, etc.), therefore we applied no filter beforehand.

A General Linear Model (GLM) analysis performed on round-level fNIRS HbO and HbR data obtained ‘beta' values, describing the goodness-of-fit of observed brain activity to an expected hemodynamic response function (HRF). For detailed information about fNIRS analyses using the GLM, please see Barker et al. (2013); Tak and Ye (2014), and Yücel et al. (2021). The GLM was fit to each NIRS channel individually per participant, resulting in one beta value for each round-participant-channel combination. HbO beta values have a positive relationship with brain activity, while HbR values have a negative relationship with brain activity. When both values are anti-correlated (e.g., positive HbO beta values and concurrent negative HbR beta values), there can be high confidence in the direction of effect on brain activity. Contrast analysis performed in NirsLAB reveals which brain areas significantly differed in activation across multiple conditions.

#### Do Agent Transparency and Reliability Link to Changes in Activation Across ROIs?

**Figure 4:**
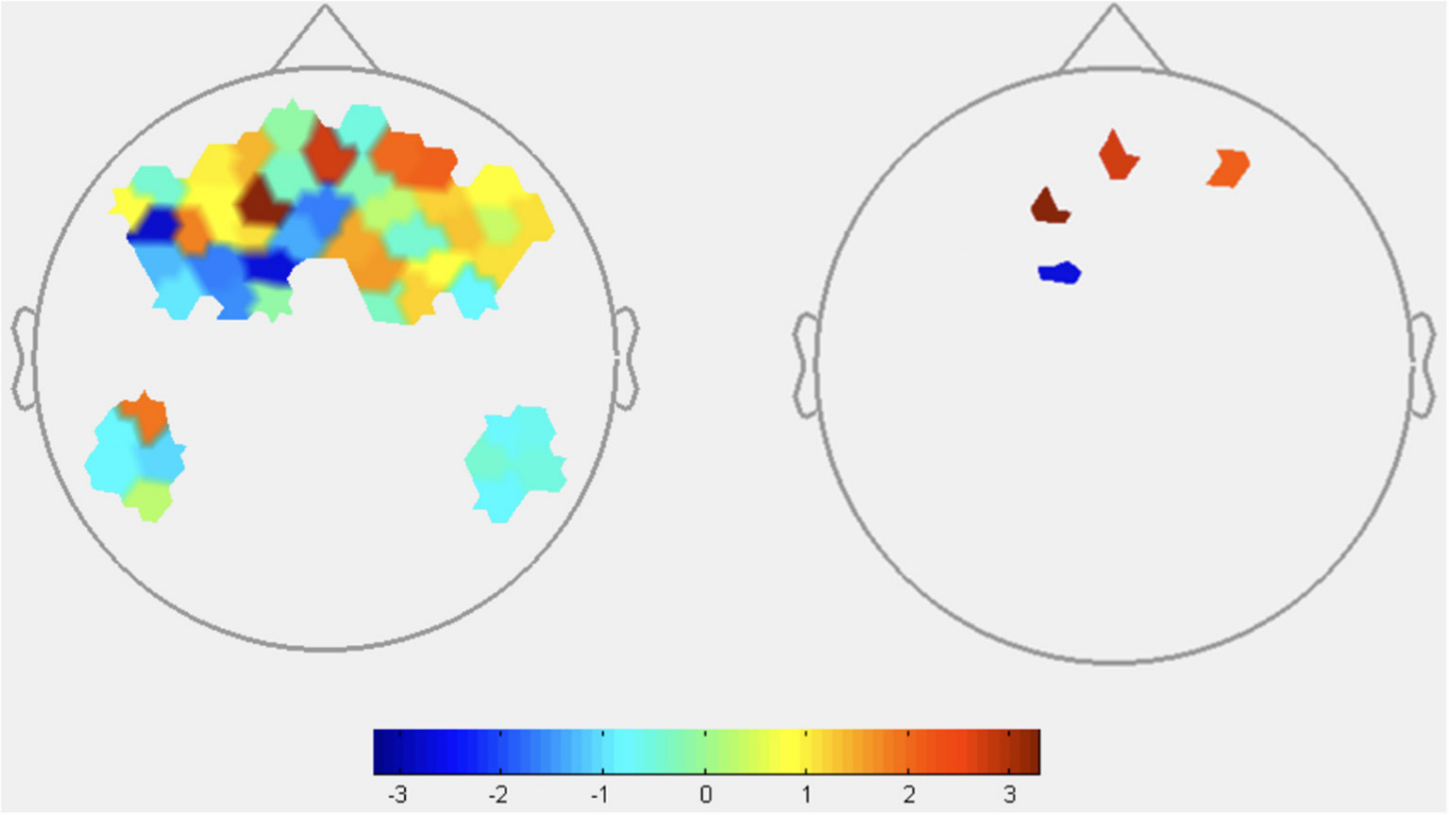
HbO contrasts between low transparency and high transparency conditions: (T^Low^R^Low^+T^low^R^High^)–(T^High^R^Low^+T^High^R^High^). Left image displays all t-values, right image displays t-values that are statistically significant (*p* < 0.05). Red coloration indicates more activation (higher HbO) during low transparency conditions compared to high transparency. Conversely, blue indicates more activation (lower HbO) during high transparency conditions.

**Figure 5:**
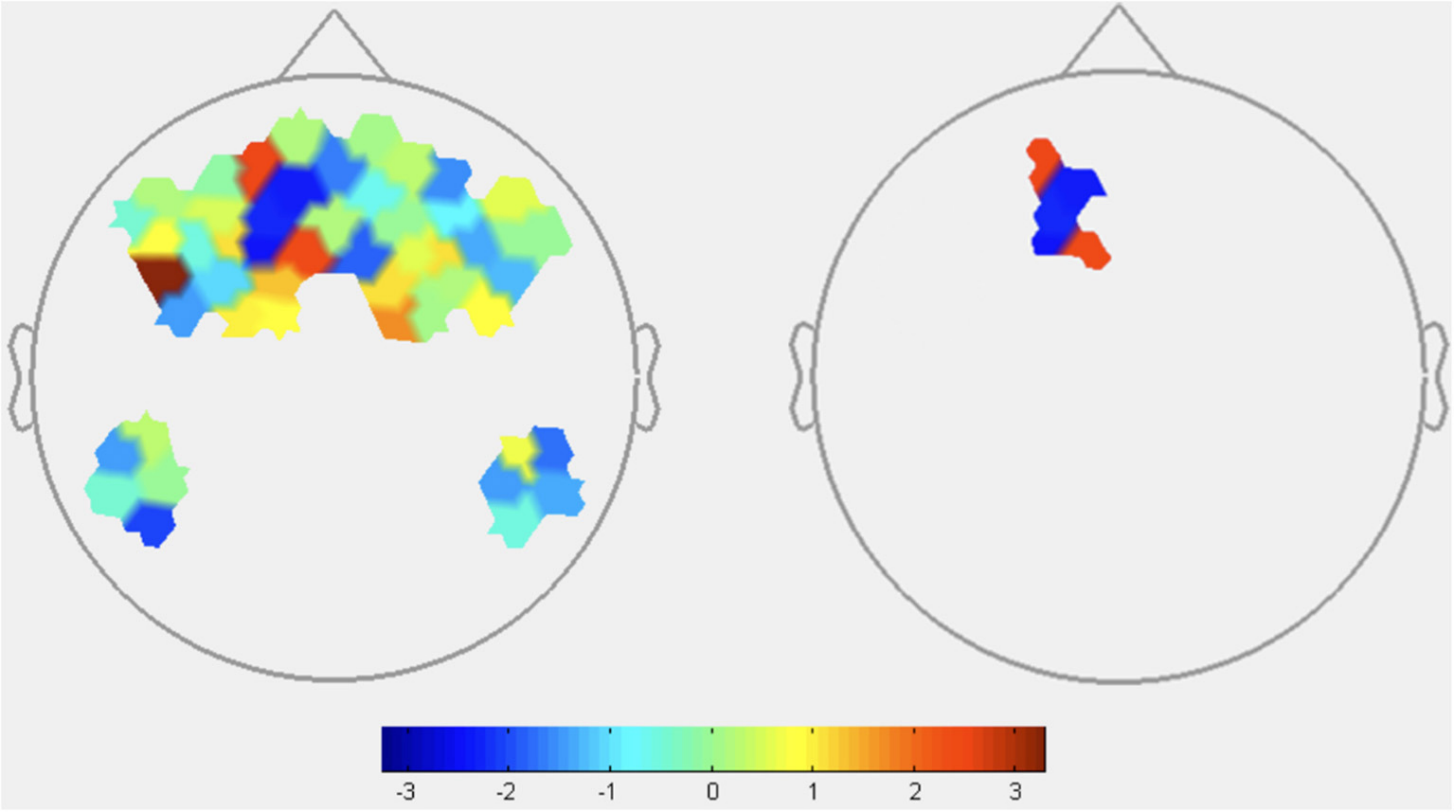
HbR contrasts between low transparency and high transparency conditions: (T^Low^R^Low^+T^low^R^High^)–(T^High^R^Low^+T^High^R^High^). Left image displays all t-values, right image displays t-values that are statistically significant (*p* < 0.05). Red coloration indicates less activation (higher HbR) during low transparency conditions. Blue coloration indicates more activation (lower HbR) during high transparency conditions.

**Figure 6:**
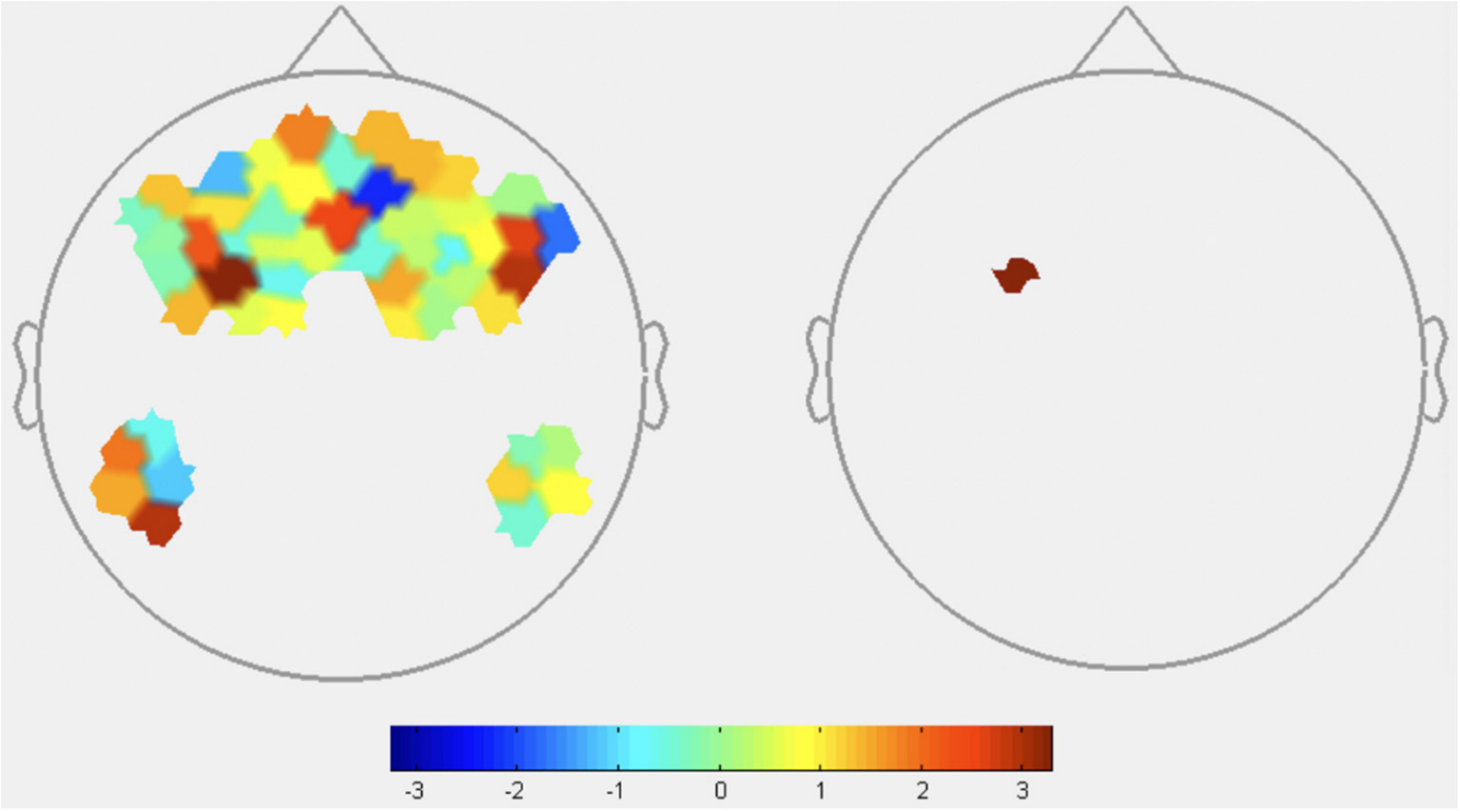
HbO contrasts between low reliability and high reliability conditions: (T^Low^R^Low^+T^High^R^Low^)–(T^Low^R^High^+T^High^R^High^). Left image displays all *t*-values, right image displays t-values that are statistically significant (*p* < 0.05). Red coloration indicates more activation (higher HbO) during low reliability conditions compared to high reliability. Conversely, blue indicates more activation (lower HbO) during high reliability conditions.

**Figure 7:**
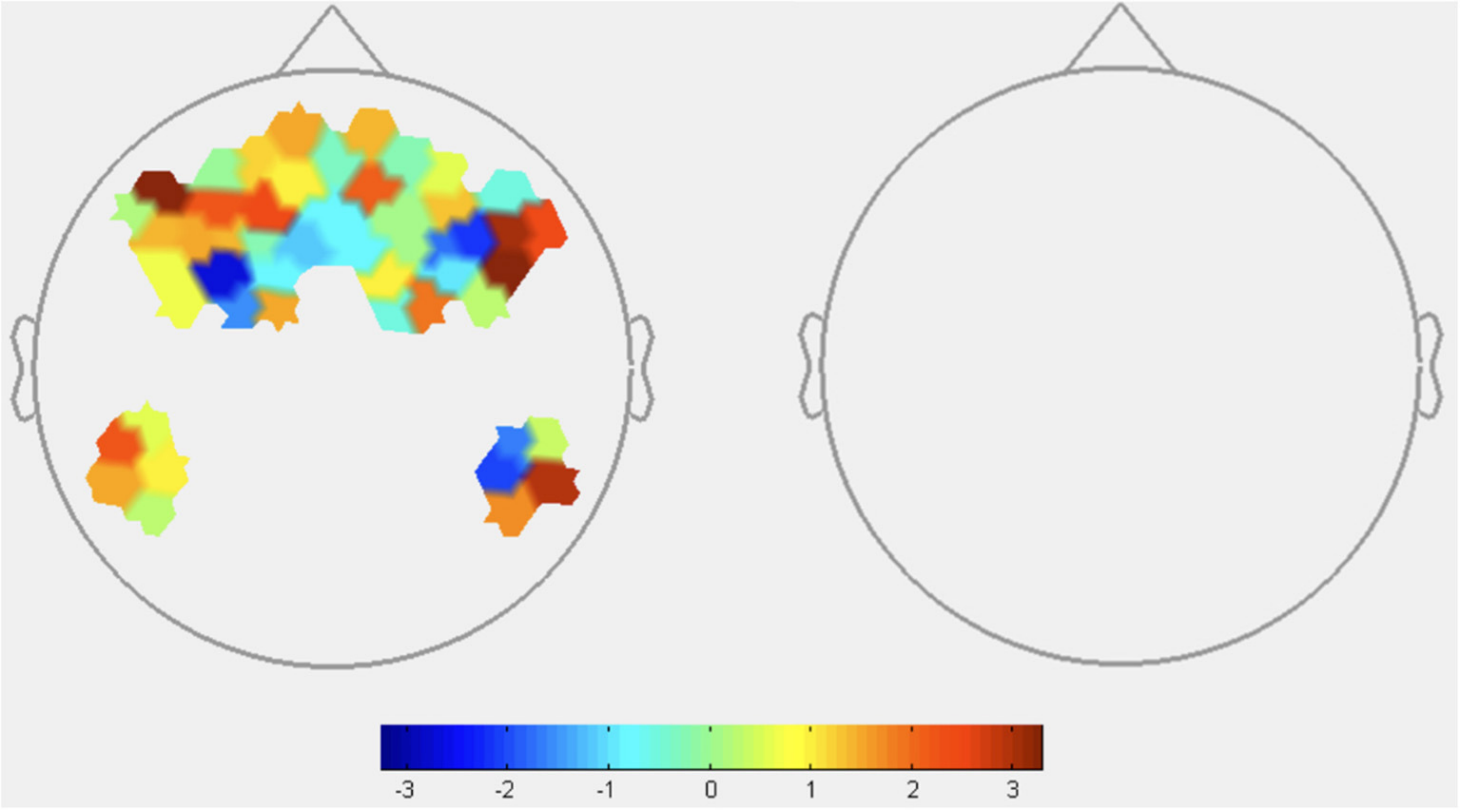
HbR contrasts between low reliability and high reliability conditions: (T^Low^R^Low^+T^High^R^Low^)–(T^Low^R^High^+T^High^R^High^). This image displays all *t*-values as there were no channels that were statistically significant (*p* < 0.05). Red coloration indicates less activation (higher HbR) during low reliability conditions. Blue coloration indicates more activation (lower HbR) during high reliability conditions.

Main effect comparisons were performed to evaluate the effects of transparency and reliability on HbO and HbR (**Figures 4**–**7** and **Tables 3**–**5**). Contrasts identify the difference in activation between conditions, with positive HbO values indicating greater activation during low transparency or reliability compared to high transparency/reliability conditions. Conversely, positive values in HbR contrasts denote decreased activation in low vs. high conditions.

##### Transparency Contrasts

HbO: As shown in Figure 4, the contrast of [transparency^low^-transparency^high^] resulted in significant HbO increases, suggestive of increased brain activation, in two channels located in the frontopolar region (see Table 3 for specific statistical values). The contrast also resulted in one channel of deactivation (e.g., decreased HbO) in the DMPFC.

**Table 3:** Oxyhemoglobin contrast results between GLM output of low transparency and high transparency conditions (T^Low^R^Low^+T^low^R^High^)-(T^High^R^Low^+T^High^R^High^).

**Channels (*p* < 0.05)**	**ROI**	**T-stat value**
4	DLPFC	3.2763
13	DMPFC	−2.7420
25	Frontopolar	2.0744
49	Frontopolar	2.6822

*Significant channel number, assigned region of interest, and t-statistic as reported by NirsLab GLM contrast analyses*.

HbR: HbR signaled increased activation in DLPFC during low transparency conditions, consistent with HbO results and supporting H1 (Figure 5 and Table 4). FPA channels primarily showed greater activity in low transparency conditions, with HbR indicating deactivation of one channel. Consistent deactivation of DMPFC channels was revealed by both HbO and HbR during low transparency.

**Table 4:** Deoxyhemoglobin contrast results between GLM output of low transparency and high transparency conditions (T^Low^R^Low^+T^low^R^High^)-(T^High^R^Low^+T^High^R^High^).

**Channels (*p* < 0.05)**	**ROI**	**T-stat value**
1	Frontopolar	2.3522
2	Frontopolar	−2.2729
4	DLPFC	−2.1098
7	DLPFC	−2.3038
11	DMPFC	2.2722

*Significant channel number, assigned region of interest, and t-statistic as reported by NirsLab GLM contrast analyses*.

From these results, omission of agent explanations caused increases in DLPFC and some FPA channels. HbO and HbR both indicate that dMPFC was more active when transparency explanations were present.

##### Reliability Contrasts

HbO: Reliability was found to significantly affect only 1 channel (Figure 6, Table 5). HbO contrasts showed increased DLPFC activity under low reliability, consistent with the expected increase in mental demand in H2.

**Table 5:** Oxyhemoglobin contrast results between GLM output of low reliability and high reliability conditions (T^Low^R^Low^+T^High^R^Low^)-(T^Low^R^High^+T^High^R^High^).

**Channels (*p* < 0.05)**	**ROI**	**T-stat value**
9	DLPFC	2.1534

*Significant channel number, assigned region of interest, and t-statistic as reported by NirsLab GLM contrast analyses*.

HbR: No significant difference in HbR was detected between reliability conditions, as seen in Figure 7.

#### Can Short Term Measures of Brain Activation Be Used to Predict Event-Level Trust in the Agent?

Our next research question pertained to whether we can identify neural correlates of participant trust in the agent on a shorter timescale. In other words, can fNIRS data be used in real-time machine learning models to predict whether or not a human is likely to rely on an AI suggestion? If an intelligent system knows with high confidence that a human user is likely (or unlikely) to rely on an agent's recent decision, then it may be desirable to prompt the human accordingly (e.g., “Don't forget! The agent is not very confident in this most recent decision, but it was confident last time.”). To address this, we extracted fNIRS data from the window of time between the agent placing a pin and a participant moving the pin (rejecting its placement) of 75 timeframes (~15 s). As a reminder, after the agent placed a pin, the humans were able to decide whether to accept the pin placement. Our behavioral logs of reliance and response time provided behavioral measures of whether the participants moved (or did not move) the most recent pin placement, and how long it took them to move a pin (if they elected to move the pin), respectively. For a proper baseline comparison, we restricted our data to instances of two possible events: (1) the agent places a pin and a participant moves that pin *before* the agent places its next pin; (2) the agent places a pin, no pin is moved before the agent places its next pin, and the agent's pin is *never* moved during that round. In other words, we directly compared brain activity between cases where participants rejected and accepted the agent's placement.

Brain data from 15 s leading up to but not including the participant moving a pin (distrust case) were compared with data from equivalent intervals where the pin was accepted and never moved (trust case). Trust events for each participant were matched with an equal number of distrust events from the same individual. As not every participant exhibited the same number of events fitting these constraints, we first averaged each individual's trust and distrust time series to ensure that individuals were not disproportionately represented in analyses. For each ROI, paired sample *t*-tests between trust and distrust events were then performed for HbO/HbR averaged across the 15 s of each event (resulting in one data point per participant per event).

**Figure 8:**
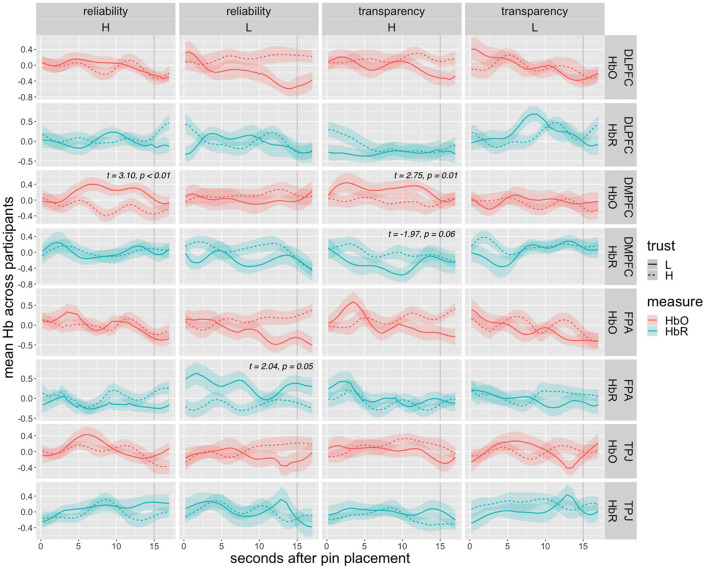
Average HbO and HbR time series after agent pin placement and before the pin was moved (distrust). Dashed lines represent trust (pin never moved) cases, solid lines represent distrust (pin moved) cases, and ribbons represent standard error range. The gray vertical line marks the moment at which the pin was moved (for distrust cases) at 15 s. Results of paired-sample *t*-test are given for significant or marginally significant ROIs.

Figure 8 presents averaged oxy- and deoxyhemoglobin time series across all participant's trust and distrust cases per ROI, along with results of paired sample *t*-tests for significant or marginally significant ROIs. Significant differences are seen under high transparency for DMPFC HbO (*t* = 2.75, *p* = 0.01) and marginally for HbR (*t* = −1.97, *p* = 0.06), under low reliability for FPA HbR (*t* = 2.04, *p* = 0.05), and under high reliability for DMPFC HbO (*t* = 3.10, *p* < 0.01).

Graphs of average HbO and HbR time series show generally higher levels of DMPFC activation leading up to the pin movement in low trust scenarios. Specifically, mean HbO was significantly higher in distrust cases under high transparency and high reliability conditions. Mean HbR supports this trend with a marginally significant effect under high transparency. Lastly, HbR indicated lower FPA activation associated with distrust in low reliability conditions.

## Discussion

We presented initial results validating and investigating the behavioral and neural outcomes of an ongoing novel, open-ended, multi-human agent teaming study. Our aim is to understand how HATs can be improved by uncovering how agent transparency and reliability affect human teammates' trust and its neural correlates.

### Findings

#### Experimental and Behavioral Findings

We used linear mixed-effects modeling to reveal the main effects of transparency and reliability and self-reported and behavioral measures of trust, reliance, mental demand, perceived performance, and affect. Controlling for time (round) and individual differences with random intercepts for teams and participants, both the transparency and reliability manipulations were found to have a nearly equivalent impact on trust. As expected, regression results indicate that the agent's performance and explanations both played a role in fostering humans' trust. However, the non-significant effect on reliance (number of agent pins accepted) suggests that the transparency and reliability manipulations affected users' internal (self-reported) trust perceptions but did not have a strong effect on their trusting actions (accepting the agent's pin) during the task. This could be due to the added interpersonal dynamics of our study vs. previous HAT experiments. In teams consisting of two humans and one agent, individuals have more sources of information and communication than when they are working solely with the agent. Having multiple humans working toward a shared reward requires a shared judgement process, as both participants must generally agree on their decision to accept or reject the agent's pins. Thus, it must be considered when interpreting these results that effects depend on one's human *and* agent teammates. This dependence may reduce the sensitivity of our reliance metric, which could also explain the lack of effect on reliance.

Notably, we found that agent transparency did influence interpersonal measures, with participants reporting lower trust in their human teammate and lower team cohesion when transparency was low. We hypothesize that this effect results from a team-level degradation of communication. Low transparency removes much of the agent's ability to communicate, which seems to weaken the team's sense of cohesion and ability to coordinate (both items assessed in the team processes survey). Based on collaborative problem solving and dynamical systems literature, this effect is to be expected in a team of three humans: in a highly collaborative task like the present study, effective teaming requires parties to share their individual knowledge to establish a common ground, monitor progress, and develop joint solutions (Roschelle and Teasley, 1995; Eloy et al., 2019; Sun et al., 2020; Stewart et al., 2021). Disrupting necessary communication dampens these emergent team-level processes, hindering performance outcomes (Eloy et al., 2019; Stewart et al., 2019). This phenomenon, however, had not yet been shown to carry over in hybrid HAT contexts. Our results provide evidence that similar dynamic processes may arise in HATs, as changes in the agent's communication behavior impacted human teammates' perceptions of each other and the team as a whole. Further work confirming this effect may be key to development of effective assistive agents that maintain team cohesion in hybrid multiparty HATs.

Low reliability conditions alone resulted in significantly slower response time, a behavioral sign linked to higher mental demand during the task. Survey scores support this finding, showing a marginally significant trend of increased mental demand in low reliability conditions. Subjects also perceived their team as less successful in accomplishing their goal in this condition. These results intuitively indicate increased task difficulty and mental demand when the agent behaves unreliably. Additionally, they confirm our expectation that the agent's delivery of transparency information does not increase mental demand as has been reported when transparency explanations are complex to interpret. Lastly, low reliability significantly degraded emotional valence. This rise in negative feelings may be linked to the increase in task difficulty and mental demand caused by an unreliable agent. We also note that we did not see an increase in arousal often associated with an increase in mental demand during trust judgements (Chen et al., 2011, p. 211; Hu et al., 2016; Hirshfield et al., 2019).

Further analysis did not reveal any significant interaction effects between transparency and reliability; the data shows that transparency and reliability had a similar, additive effect on reported trust when manipulated together. More extensive modeling procedures are required to piece apart the linear and non-linear effects of time and its potential interaction with the manipulations. Effects of manipulations in HAT studies are generally very context-dependent due to task differences, emphasizing the importance of considering these initial trends when interpreting the following analyses and comparing with other studies. With this in mind, we examine fNIRS results with an understanding of the saliency of transparency and reliability manipulations on agent trust, mental demand, affective valence, interpersonal trust, and team processes.

#### Block-Level fNIRS Contrasts

Block-level Oxyhemoglobin contrast analyses highlighted brain regions sensitive to agent transparency and reliability. Contrasts between transparency conditions revealed significant DLPFC and FPA activation and significant DMPFC deactivation in low transparency conditions. Deoxyhemoglobin contrasts provide mixed results with no consistent trend across the 2 significant FPA channels. However, HbR does show activation in DLPFC and deactivation in DMPFC congruent with HbO results. While HbR is generally less understood and relied on in fNIRS literature, it is noteworthy to acknowledge that low transparency results in greater activation for both HbO and HbR in the DLPFC and part of the FPA regions, suggesting an interconnectivity between the two regions when transparency is low.

FPA and DLPFC activity is associated with high-workload task processing, which aligns with consistent activation during low transparency as participants had to independently process information for credibility assessments due to the lack of agent explanations. This is also consistent with prior fNIRS research implicating FPA and DLPFC in a state of suspended judgement and increased mental demand when trying to determine veracity of a teammate's recommendation (Hirshfield et al., 2019; Palmer et al., 2019).

Based on existing functional research, less activation of DMPFC in low transparency compared to high transparency conditions, as seen in Oxy- and HbR contrasts, implies a reduction in social reasoning and judgements about others. The lack of information in low transparency conditions could explain this effect, suggesting DMPFC was recruited specifically when processing information given by the agent to judge its intent and credibility. When transparency information is missing, individuals in a state of suspended judgement rely more heavily on their *own* knowledge as opposed to the agent's. Thus, when transparency information was present, participants may have needed greater DMPFC activity to process and reason about information provided by the agent.

Contrasts of reliability conditions only identified one significant channel, corresponding to DLPFC HbO. This effect is no surprise given the evidence of DLPFC activation in periods of higher cognitive load (McKendrick and Harwood, 2019) and increased suspicion (Hirshfield et al., 2019). As discussed under Experimental and Behavioral Findings, low reliability corresponded to decreased trust and increased response time, self-reported mental demand, and perceived task difficulty. When the agent is unreliable and is performing poorly, participants must perform trustworthiness assessments of the AI pin placements. This could be responsible for increased DLPFC activation as reliability was manipulated. Increased activation also points to the DLPFC's role in regulation of negative emotion (Yang et al., 2016), as survey results linked low reliability conditions to negative valence. However, further work is needed to specify these effects and better characterize this pattern of DLPFC activation.

Regarding the lack of reliability-sensitive channels compared to transparency contrasts, we note that participants were only presented with their team's and the agent's scores at the end of each round. As a result, gauging reliability requires more careful evaluation of the agent's recommendations during each round. This may explain why we observe a less salient difference in block-level brain activation between reliability conditions compared to transparency. This might also explain why reliability effects are more noticeable in survey results, as fNIRS data is recorded during each round as opposed to surveys taken after receiving performance scores.

These contrasts allow us to begin piecing apart the large-scale effects of transparency and reliability on neural activity. Based on our results, it appears that decreasing the agent's trustworthiness and subsequently increasing mental demand through transparency and reliability manipulations successfully recruited DLPFC activation. While both manipulations were linked to neural activations, transparency contrasts suggest a nuanced role of DMPFC in judging information provided by the agent. These results identify potential neural correlates of human-agent trust under different conditions, simulating a realistic collaboration scenario that could benefit from applications in neuroergonomics of intelligent trust sensing and modulation. As we discuss potential functional explanations for our fNIRS results, we reiterate that more studies are needed to confirm and further specify these effects.

### Event-Level Neural Correlates for Real-Time Trust Prediction

We dissected event-level instances of trust to narrow in on the large-scale effects seen in round-level analyses. We believe our initial results are compelling, showing that activity in DMPFC varied between high and low trust decisions in some conditions. Notably, mean HbO and HbR (marginally significant) in DMPFC indicated higher activity before low trust events in high transparency conditions. This increased activation may be a signal of increased skepticism as the participant makes a judgement about the agent's trustworthiness based on information provided. In our block-level contrast results HbO and HbR also showed increased DMPFC activity in high transparency conditions as it was recruited to process the agent's explanations when making trust decisions. Event-level results support this claim by suggesting that if the agent gives an explanation, the DMPFC is more active when the individual ultimately decides the agent's placement is not trustworthy. The same significant effect was also seen under high reliability conditions. A potential interpretation of this effect is that under high reliability, individuals are aware that the agent *might* be trustworthy and must more carefully consider the details of the agent's suggestion. Because teams work toward a shared reward, accepting the agent's pin placements could seem riskier to an individual than moving the pin to another location of their choosing. In this case, the reliability of the agent's previous pin placements (as opposed to explanations in the high transparency case) is the information that must be considered when deciding to accept or reject the current pin.

FPA HbR was also a significant predictor of trust in low reliability conditions, with less FPA activation in low trust cases. Although block-level results showed no significant FPA channels, this effect does appear to contradict what we might expect given that low trust generally equated to higher mental demand. The difference in event-level activation suggests that when the agent clearly performed poorly, FPA was significantly less active in making trust decisions when the pin was rejected. Considering the expected increase in cognitive load from low reliability conditions, this could indicate task overload, where a drop in activity, accompanied by a drop in performance, is observed following a high mental demand threshold (Bunce et al., 2011).

A trust-sensitive adaptive system must be able to assess the state of the environment while processing neurophysiological sensor data to choose the most appropriate action. If the operator of a self-driving vehicle is manually driving on an unfamiliar road in a state of low trust and high mental demand, any communication or suggestion from the vehicle might overload the driver, hindering performance and damaging future trust even if the vehicle has high confidence. A system's reliability, transparency, and certainty level, among other factors, not only determine the driver's current state of trust, but also determine how the system's next action will be received.

### Applications and Considerations in Neuroergonomics

The findings presented in this paper begin to detangle the neural dynamics of trust and teaming in an open-ended collaborative task of 2 humans with 1 agent. fNIRS devices are particularly suited for ambulatory applications and online analysis, and we believe real-time data at both the block-level and event-level can realistically be used to predict states of human trust and mental demand. As HATs become more common and accessible, we will begin to see more hybrid teams of multiple humans with one agent, or multiple humans with multiple agents. These systems will therefore need to adapt to monitor states of multiple individuals along with emergent team-level states under different environmental constraints. Identifying significant markers of trust and team processes, such as DLPFC and DMPFC, in different agent behavior contexts is a vital step to building adaptive systems to improve efficiency performance of human-agent collaborations.

As our results demonstrated, changes in agent behavior can invert or suppress an expected effect. To generalize across individuals and their ever-changing environments, trust-sensitive systems must monitor and adapt to these shifting contexts to maximize team performance. For example (in the context of this study), an adaptive extension of the CHART assistive agent might sense increased DLPFC activation in its teammates, recognize this as high mental demand, and begin suggesting pin placements with explanations to encourage its teammates to offload tasks onto it. If the agent subsequently detects a spike in DMPFC HbO following pin placements, the agent might predict that its teammates are in a low trust state and are performing taxing trustworthiness assessments of each pin, prompting the agent to reconsider its method of explanation. Indeed, actions and interventions must be carefully considered in HAT implementations to optimize cognitive load while maintaining performance, engagement, and comfort. To that end, we direct readers to a recent paper (Dehais et al., 2020) proposing a dynamic model of human workload and performance, as determined by arousal and task engagement. We believe adaptive systems designed to work toward a “comfort zone” of workload will more effectively promote productive, extended collaborations by avoiding human overload or disengagement over time. Coupling this model with an awareness of trust will result in a flexible system capable of adapting to and appropriately tuning the social, cognitive, and affective states of a team to fully utilize the computational power and contribution of assistive agents.

We aim (and encourage others) to extend the foundation of results in this article to begin implementation of a real-time adaptive trust-modulation system built on a reinforcement learning framework. While beyond the scope of this initial paper, we want to highlight the potential of existing mathematical frameworks in complex decision-making applications. Specifically, partially-observable Markov decision process (POMDP) models are well-equipped to deal with the inherent uncertainty of human behavior, and are thus gaining traction in human-computer interaction research. POMDPs assume underlying states are not directly measurable, instead considering probabilities of state transitions given the agent's actions as well as a history of states. The model is given a reward function, rewarding or penalizing certain state transitions, and learns to choose the action at a given state that maximizes expected rewards. The CHART agent in the example above might then be rewarded when human teammates are in the “comfort zone” of workload and trust levels are calibrated to match the agent's certainty level.

With any process attempting to classify human states with sensor observations, inherent ambiguities arise due to individual differences, histories, high-dimensional interpersonal social dynamics, the multi-faceted nature of trust, and our incomplete understanding of the human brain and neural circuitry. For this reason, POMDPs and similar models are gaining increasing attention for their built-in assumptions of uncertainty. We highlight this general approach because individually accounting for these countless complexities would be intractable and may reduce generalizability. As collaborative HAT systems become more sophisticated, it is increasingly important for researchers and designers to account for these uncertainties to improve effectiveness and appropriately interpret effects. As we continue collecting data and identifying neurophysiological markers of trust, mental demand, and other states, we aim to develop a robustly adaptive agent that can learn to promote effective human-agent collaborations across a variety of contexts.

### Limitations and Future Work

Our work has six main limitations to be addressed in the future. First, we address experimental limitations that arise due to the length of the study. The NIRx sensors, while non-invasive, can cause discomfort over long periods due to the tightness of the headcap and pressure from the optode probes against the scalp. During initial pilot studies we found 2 h to be the maximum amount of time participants could comfortably perform the task. Due to the tradeoff between time spent in the task with fNIRS sensors and time spent on post-task surveys, we used only the top factor loading items to assess constructs of interest across 8 full rounds. We acknowledge that paring down surveys can reduce interpretability, and that future work should leverage a more robust set of surveys measures to better disentangle observed effects. Similarly, behavioral measures of reliance and response time indirectly measure latent variables like trust and workload, which limits what conclusions can be drawn. Next, our study is an ongoing multimodal data collection effort at a single university, so results presented in this article are limited in sample size and demographic representation. Third, processing large data sets from multiple sensors (galvanic skin response, eye-tracking, and speech data are recorded along with NIRS data during the task) is time-consuming and computationally demanding, so we constrained the scope of this article to main effects of one modality to allow time for analysis. In particular, we note that event-level paired sample *t*-tests were performed on a restricted (undersampled majority class) dataset, and future efforts to apply machine learning will better deal with this class imbalance for a more robust dataset. Fourth, we have yet to investigate the rich dynamics of trust development over time. Results of mixed-effects models of behavioral and survey measures are limited to presenting high-level trends, as no model selection procedure or further modeling of the effects of time are presented in this article. Fifth, although changes in agent behavior manipulated human-agent trust, only one task is examined, which limits generalizability claims. Lastly, our analyses focus primarily on the individual and therefore do not fully capture the rich, high-dimensional dynamics that arise when considering teams as a single system. Implementing team-level analyses is a clear next step in our investigation of trust and the social dynamics of HATs. These limitations combined with the complex, open-ended task introduce many uncertainties inherent to the ambitious fields of neuroergonomics and human-computer interaction. We hope that with further research we can address these uncertainties and narrow in on the specific effects presented in this article.

Given this ambition and complexity, we believe our results are compelling and provide an exciting direction for future studies. We are continuing to process data to begin multimodal team-level analyses to further identify markers of trust and mental demand throughout the collaboration. Training machine learning models to predict trust/distrust events from fNIRS recordings is a concrete next step toward real-time trust monitoring. Future work should also strive to model human-agent teams across a variety of open-ended tasks and settings. Additionally, developing effective trust modulation systems will require a rich understanding of trust degradation and trust repair over time (De Visser et al., 2018). As this literature grows, the research community will gain an increasingly detailed understanding of neural processes that govern complex social interactions with agents, as well as the nuanced effects that arise in different contexts.

## Data Availability Statement

The raw data supporting the conclusions of this article will be made available by the authors, without undue reservation.

## Ethics Statement

The studies involving human participants were reviewed and approved by University of Colorado Boulder Institutional Review Board. The patients/participants provided their written informed consent to participate in this study.

## Author Contributions

LE led experimental design, oversaw data collection, selected and carried out analysis of behavioral and non-GLM fNIRS data, and drafted most of the text in the article. ED configured fNIRS devices, assisted in selecting analyses, and carried out all GLM contrast analyses of fNIRS data, as well as filtering and exporting data, and assisted in writing and interpretation of results. CS assisted in extraction and analysis of behavioral and survey data, writing of the introduction and interpretations, and was responsible for data checks and replicating analyses. PB (secondary senior author) assisted in theory development and experimental design, provided guidance on analyses, and guided drafting of introduction and discussion text. LH (senior author) is PI on this study's grant, leader of the lab, and oversaw and directed design of the study, development of the testbed, and drafting the article. All authors contributed to the article and approved the submitted version.

## Funding

Army Research Office is fully funding all research efforts.

## Author Disclaimer

The views, opinions, and/or findings contained in this article are those of the authors and should not be interpreted as representing the official views or policies, either expressed or implied, of the funding agencies.

## Conflict of Interest

The authors declare that the research was conducted in the absence of any commercial or financial relationships that could be construed as a potential conflict of interest.

## Publisher's Note

All claims expressed in this article are solely those of the authors and do not necessarily represent those of their affiliated organizations, or those of the publisher, the editors and the reviewers. Any product that may be evaluated in this article, or claim that may be made by its manufacturer, is not guaranteed or endorsed by the publisher.

## References

[B1] Aimone J. A. Houser D. Weber B. (2014). Neural signatures of betrayal aversion: an fMRI study of trust. Proc. Royal Soc. 281:20132127. 10.1098/rspb.2013.2127 24648217 PMC3973250

[B2] Akash K. Hu W.-L. Jain N. Reid T. (2018). A classification model for sensing human trust in machines using EEG and GSR. ACM Transac. Interactive Intelligent Syst. 8, 1–20. 10.1145/3132743

[B3] Akash K. McMahon G. Reid T. Jain N. (2020). Human trust-based feedback control: dynamically varying automation transparency to optimize human-machine interactions. IEEE Control Syst. Magazine 40, 98–116. 10.1109/MCS.2020.3019151

[B4] Ayaz H. Shewokis P. A. Bunce S. Izzetoglu K. Willems B. Onaral B. (2012). Optical brain monitoring for operator training and mental workload assessment. Neuroimage 59, 36–47. 10.1016/j.neuroimage.2011.06.023 21722738

[B5] Barker J. W. Aarabi A. Huppert T. J. (2013). Autoregressive model based algorithm for correcting motion and serially correlated errors in fNIRS. Biomed. Optics Express 4, 1366–1379. 10.1364/BOE.4.001366 24009999 PMC3756568

[B6] Bates D. Mächler M. Bolker B. Walker S. (2015). Fitting linear mixed-effects models using lme4. J. Statistical Softw. 67, 1–48. 10.18637/jss.v067.i01

[B7] Bhaskara A. Skinner M. Loft S. (2020). Agent transparency: a review of current theory and evidence. IEEE Transac. Human-Machine Syst. 50, 215–224. 10.1109/THMS.2020.2965529

[B8] Bhatt M. A. Lohrenz T. Camerer C. F. Montague P. R. (2012). Distinct contributions of the amygdala and parahippocampal gyrus to suspicion in a repeated bargaining game. Proc. Natl. Acad. Sci. U.S.A. 109, 8728–8733. 10.1073/pnas.1200738109 22582170 PMC3365181

[B9] Bunce S. C. Izzetoglu K. Ayaz H. Shewokis P. Izzetoglu M. Pourrezaei K.. (2011). “Implementation of fNIRS for monitoring levels of expertise and mental workload,” in International Conference on Foundations of Augmented Cognition (Orlando, FL), 13–22. 10.1007/978-3-642-21852-1_2

[B10] Chancey E. T. Bliss J. P. Proaps A. B. Madhavan P. (2015). The role of trust as a mediator between system characteristics and response behaviors. Human Factors 57, 947–958. 10.1177/0018720815582261 25917611

[B11] Chen J. Y. Barnes M. J. Kenny C. (2011). “Effects of unreliable automation and individual differences on supervisory control of multiple ground robots,” in 2011 6th ACM/IEEE International Conference on Human-Robot Interaction (HRI) (Lausanne: IEEE), 371–378. 10.1145/1957656.1957793

[B12] Chiou E. K. Lee J. D. (2021). Trusting automation: designing for responsivity and resilience. Human Factors. 1–29. 10.1177/00187208211009995 33906505

[B13] Curtin A. Ayaz H. (2018). The age of neuroergonomics: towards ubiquitous and continuous measurement of brain function with fNIRS. Jap. Psychol. Res. 60, 374–386. 10.1111/jpr.12227

[B14] De Visser E. J. Pak R. Shaw T. H. (2018). From ‘automation'to ‘autonomy': The importance of trust repair in human–machine interaction. Ergonomics 61, 1409–1427. 10.1080/00140139.2018.1457725 29578376

[B15] DeCostanza A. H. Marathe A. R. Bohannon A. Evans A. W. Palazzolo E. T. Metcalfe J. S.. (2018). Enhancing humanagent teaming with individualized, adaptive technologies: A discussion of critical scientific questions. US Army Research Laboratory Aberdeen Proving Ground United States.

[B16] Dehais F. Lafont A. Roy R. Fairclough S. (2020). A neuroergonomics approach to mental workload, engagement and human performance. Front. Neurosci. 14:268. 10.3389/fnins.2020.00268 32317914 PMC7154497

[B17] Denny B. T. Kober H. Wager T. D. Ochsner K. N. (2012). A meta-analysis of functional neuroimaging studies of self-and other judgments reveals a spatial gradient for mentalizing in medial prefrontal cortex. J. Cogn. Neurosci. 24, 1742–1752. 10.1162/jocn_a_00233 22452556 PMC3806720

[B18] Dimoka A. (2010). What does the brain tell us about trust and distrust? Evidence from a functional neuroimaging study. Mis Quart. 34, 373–396. 10.2307/20721433

[B19] Durantin G. Gagnon J.-F. Tremblay S. Dehais F. (2014). Using near infrared spectroscopy and heart rate variability to detect mental overload. Behav. Brain Res. 259, 16–23. 10.1016/j.bbr.2013.10.042 24184083

[B20] Eloy L. E. B. Stewart A. Jean Amon M. Reinhardt C. Michaels A. Sun C.. (2019). “Modeling team-level multimodal dynamics during multiparty collaboration,” in 2019 International Conference on Multimodal Interaction (Suzhou), 244–258. 10.1145/3340555.3353748

[B21] Fett A.-K. J. Gromann P. M. Giampietro V. Shergill S. S. Krabbendam L. (2014). Default distrust? An fMRI investigation of the neural development of trust and cooperation. Soc. Cognitive Affective Neurosci. 9, 395–402. 10.1093/scan/nss144 23202661 PMC3989120

[B22] Filkowski M. M. Anderson I. W. Haas B. W. (2016). Trying to trust: brain activity during interpersonal social attitude change. Cognitive Affective Behav. Neurosci. 16, 325–338. 10.3758/s13415-015-0393-0 26567160

[B23] Glikson E. Woolley A. W. (2020). Human trust in artificial intelligence: review of empirical research. Acad. Manage. Annals 14, 627–660. 10.5465/annals.2018.005732454901

[B24] Gupta K. Hajika R. Pai Y. S. Duenser A. Lochner M. Billinghurst M. (2019). “In ai we trust: Investigating the relationship between biosignals, trust and cognitive load in vr,” in 25th ACM Symposium on Virtual Reality Software and Technology, 1–10. 10.1145/3359996.3364276

[B25] Gvozdenko E. Chambers D. (2007). Beyond test accuracy: benefits of measuring response time in computerised testing. Austral. J. Educ. Tech. 23, 542–558. 10.14742/ajet.1251

[B26] Hagras H. (2018). Toward human-understandable, explainable AI. Computer 51, 28–36. 10.1109/MC.2018.362096534467649

[B27] Hawkins K. A. Fox E. J. Daly J. J. Rose D. K. Christou E. A. McGuirk T. E.. (2018). Prefrontal over-activation during walking in people with mobility deficits: Interpretation and functional implications. Hum. Mov. Sci. 59, 46–55. 10.1016/j.humov.2018.03.010 29604488 PMC5988641

[B28] Helldin T. (2014). Transparency for Future Semi-Automated Systems: Effects of Transparency on Operator Performance, Workload and Trust [Ph.D. Thesis]. Örebro Universitet.

[B29] Hirshfield L. Bobko P. Barelka A. Sommer N. Velipasalar S. (2019). Toward interfaces that help users identify misinformation online: using fNIRS to measure suspicion. Augmented Human Res. 4, 1–13. 10.1007/s41133-019-0011-8

[B30] Hoff K. A. Bashir M. (2015). Trust in automation: integrating empirical evidence on factors that influence trust. Human Factors 57, 407–434. 10.1177/0018720814547570 25875432

[B31] Hu W.-L. Akash K. Jain N. Reid T. (2016). Real-time sensing of trust in human-machine interactions^**^this material is based upon work supported by the National Science Foundation under Award No. 1548616. Any opinions, findings, and conclusions or recommendations expressed in this material are those of the author(s) and do not necessarily reflect the views of the National Science Foundation. IFAC-PapersOnLine 49, 48–53. 10.1016/j.ifacol.2016.12.188

[B32] Hussain M. S. AlZoubi O. Calvo R. A. D'Mello S. K. (2011). “Affect detection from multichannel physiology during learning sessions with AutoTutor,” in International Conference on Artificial Intelligence in Education, 131–138.

[B33] Hussein A. Elsawah S. Abbass H. A. (2020). The reliability and transparency bases of trust in human-swarm interaction: principles and implications. Ergonomics 63, 1116–1132. 10.1080/00140139.2020.1764112 32370651

[B34] Krueger F. McCabe K. Moll J. Kriegeskorte N. Zahn R. Strenziok M.. (2007). Neural correlates of trust. Proc. Natl. Acad. Sci. U.S.A. 104, 20084–20089. 10.1073/pnas.0710103104 18056800 PMC2148426

[B35] Kunze A. Summerskill S. J. Marshall R. Filtness A. J. (2019). Automation transparency: implications of uncertainty communication for human-automation interaction and interfaces. Ergonomics 62, 345–360. 10.1080/00140139.2018.1547842 30501566

[B36] Lee J. D. See K. A. (2004). Trust in automation: designing for appropriate reliance. Human Fact. 46, 50–80. 10.1518/hfes.46.1.50.30392 15151155

[B37] Liu Y. Ayaz H. Shewokis P. A. (2017). Multisubject “learning” for mental workload classification using concurrent EEG, fNIRS, and physiological measures. Front. Human Neurosci. 11:389. 10.3389/fnhum.2017.00389 28798675 PMC5529418

[B38] Madhavan P. Wiegmann D. A. (2007). Similarities and differences between human–human and human–automation trust: an integrative review. Theoretical Ergonom. Sci. 8, 277–301. 10.1080/14639220500337708

[B39] Mahy C. E. Moses L. J. Pfeifer J. H. (2014). How and where: theory-of-mind in the brain. Dev. Cogn. Neurosci. 9, 68–81. 10.1016/j.dcn.2014.01.002 24552989 PMC6989753

[B40] Marks M. A. Mathieu J. E. Zaccaro S. J. (2001). A temporally based framework and taxonomy of team processes. Acad. Manage. Rev. 26, 356–376. 10.5465/amr.2001.4845785

[B41] Mathieu J. E. Luciano M. M. D'Innocenzo L. Klock E. A. LePine J. A. (2020). The development and construct validity of a team processes survey measure. Organiz. Res. Methods 23, 399–431. 10.1177/1094428119840801

[B42] McKendrick R. Harwood A. (2019). Cognitive workload and workload transitions elicit curvilinear hemodynamics during spatial working memory. Front. Human Neurosci. 13:405. 10.3389/fnhum.2019.00405 31824274 PMC6880762

[B43] McKendrick R. D. Cherry E. (2018). A deeper look at the NASA TLX and where it falls short. Proc. Human Fact. Ergonomics Soc. Annual Meeting 62, 44–48. 10.1177/1541931218621010

[B44] Merritt S. M. (2011). Affective processes in human–automation interactions. Human Factors 53, 356–370. 10.1177/0018720811411912 21901933

[B45] Miller C. A. (2021). “Trust, transparency, explanation, and planning: why we need a lifecycle perspective on human-automation interaction,” in Trust in Human-Robot Interaction (Cambridge, MA: Elsevier), 233–257. 10.1016/B978-0-12-819472-0.00011-3

[B46] Mitchell J. P. Banaji M. R. Macrae C. N. (2005). The Link between social cognition and self-referential thought in the medial prefrontal cortex. J. Cogn. Neurosci. 17, 1306–1315. 10.1162/0898929055002418 16197685

[B47] Morais G. A. Z. Balardin J. B. Sato J. R. (2018). fNIRS optodes' location decider (fOLD): a toolbox for probe arrangement guided by brain regions-of-interest. Sci. Rep. 8:3341. 10.1038/s41598-018-21716-z 29463928 PMC5820343

[B48] Mouloua M. Hancock P. A. (2019). Human Performance in Automated and Autonomous Systems, Two-Volume Set. Boca Raton, FL: CRC Press. 10.1201/9780429458347

[B49] Nozawa T. Sasaki Y. Sakaki K. Yokoyama R. Kawashima R. (2016). Interpersonal frontopolar neural synchronization in group communication: an exploration toward fNIRS hyperscanning of natural interactions. NeuroImage 133, 484–497. 10.1016/j.neuroimage.2016.03.059 27039144

[B50] Palmer S. Richards D. Shelton-Rayner G. Inch D. Izzetoglu K. (2019). “Human-agent teaming-an evolving interaction paradigm: an innovative measure of trust,” in 20th International Symposium on Aviation Psychology 438.

[B51] Parasuraman R. Molloy R. Singh I. L. (1993). Performance consequences of automation-induced'complacency'. Int. J. Aviation Psychol. 3, 1–23. 10.1207/s15327108ijap0301_1

[B52] Parasuraman R. Riley V. (1997). Humans and automation: Use, misuse, disuse, abuse. Human Fact. 39, 230–253. 10.1518/00187209777854388618689046

[B53] Parasuraman R. Sheridan T. B. Wickens C. D. (2008). Situation awareness, mental workload, and trust in automation: viable, empirically supported cognitive engineering constructs. J. Cognitive Eng. Decision Making 2, 140–160. 10.1518/155534308X284417

[B54] Pfeifer M. D. Scholkmann F. Labruyère R. (2018). Signal processing in functional near-infrared spectroscopy (fNIRS): methodological differences lead to different statistical results. Front. Human Neurosci. 11:641. 10.3389/fnhum.2017.00641 29358912 PMC5766679

[B55] Piper S. K. Krueger A. Koch S. P. Mehnert J. Habermehl C. Steinbrink J.. (2014). A wearable multi-channel fNIRS system for brain imaging in freely moving subjects. Neuroimage 85, 64–71. 10.1016/j.neuroimage.2013.06.062 23810973 PMC3859838

[B56] Pushkarskaya H. Smithson M. Joseph J. E. Corbly C. Levy I. (2015). Neural correlates of decision-making under ambiguity and conflict. Front. Behav. Neurosci. 9:325. 10.3389/fnbeh.2015.00325 26640434 PMC4661279

[B57] Roschelle J. Teasley S. D. (1995). The construction of shared knowledge in collaborative problem solving. Computer Supported Collaborative Learn. 69–97. 10.1007/978-3-642-85098-1_534790147

[B58] Rousseau D. M. Sitkin S. B. Burt R. S. Camerer C. (1998). Not so different after all: a cross-discipline view of trust. Acad. Manage. Rev. 23, 393–404. 10.5465/amr.1998.926617

[B59] Russell J. A. (1980). A circumplex model of affect. J. Personal. Soc. Psychol. 39:1161. 10.1037/h0077714

[B60] Salazar M. Shaw D. J. Gajdo,š M. Mareček R. Czekóov,á K. Mikl M.. (2021). You took the words right out of my mouth: dual-fMRI reveals intra-and inter-personal neural processes supporting verbal interaction. NeuroImage 228:117697. 10.1016/j.neuroimage.2020.117697 33385556

[B61] Schmitz C. H. Klemer D. P. Hardin R. Katz M. S. Pei Y. Graber H. L.. (2005). Design and implementation of dynamic near-infrared optical tomographic imaging instrumentation for simultaneous dual-breast measurements. Appl. Optics 44, 2140–2153. 10.1364/AO.44.002140 15835360

[B62] Schneider P. Piper S. Schmitz C. Schreiter N. Volkwein N. Lüdemann L.. (2011). Fast 3D near-infrared breast imaging using indocyanine green for detection and characterization of breast lesions. RöFo-Fortschritte Auf Dem Gebiet Der Röntgenstrahlen Und Der Bildgebenden Verfahren 183, 956–963. 10.1055/s-0031-1281726 21972043

[B63] Sebastian C. L. Fontaine N. M. Bird G. Blakemore S.-J. De Brito S. A. McCrory E. J.. (2012). Neural processing associated with cognitive and affective theory of Mind in adolescents and adults. Soc. Cognitive Affect. Neurosci. 7, 53–63. 10.1093/scan/nsr023 21467048 PMC3252629

[B64] Stewart A. E. Keirn Z. D'Mello S. K. (2021). “Multimodal modeling of collaborative problem-solving facets in triads,” in User Modeling and User-Adapted Interaction, 1–39. 10.1007/s11257-021-09290-y

[B65] Stewart A. E. Vrzakova H. Sun C. Yonehiro J. Stone C. A. Duran N. D.. (2019). I say, you say, we say: using spoken language to model socio-cognitive processes during computer-supported collaborative problem solving. Proc. ACM Human-Computer Inte. 3, 1–19. 10.1145/3359296

[B66] Sun C. Shute V. J. Stewart A. Yonehiro J. Duran N. D'Mello S. (2020). Towards a generalized competency model of collaborative problem solving. Comput. Educ. 143:103672. 10.1016/j.compedu.2019.103672

[B67] Tak S. Ye J. C. (2014). Statistical analysis of fNIRS data: a comprehensive review. Neuroimage 85, 72–91. 10.1016/j.neuroimage.2013.06.016 23774396

[B68] Tang H. Mai X. Wang S. Zhu C. Krueger F. Liu C. (2016). Interpersonal brain synchronization in the right temporo-parietal junction during face-to-face economic exchange. Soc. Cogn. Affect. Neurosci. 11, 23–32. 10.1093/scan/nsv092 26211014 PMC4692317

[B69] Wang M. Hussein A. Rojas R. F. Shafi K. Abbass H. A. (2018). “EEG-based neural correlates of trust in human-autonomy interaction,” in 2018 IEEE Symposium Series on Computational Intelligence (SSCI), 350–357. 10.1109/SSCI.2018.8628649

[B70] Wang N. Pynadath D. V. Hill S. G. (2016). “Trust calibration within a human-robot team: comparing automatically generated explanations,” in 2016 11th ACM/IEEE International Conference on Human-Robot Interaction (HRI) (Christchurch: IEEE), 109–116. 10.1109/HRI.2016.7451741

[B71] Watabe M. Ban H. Yamamoto H. (2011). Judgments about others' trustworthiness: an fMRI study. Lett. Evolut. Behav. Sci. 2, 28–32. 10.5178/lebs.2011.16

[B72] Wright J. L. Chen J. Y. Lakhmani S. G. (2019). Agent transparency and reliability in human–robot interaction: the influence on user confidence and perceived reliability. IEEE Transac. Human-Machine Syst. 50, 254–263. 10.1109/THMS.2019.2925717

[B73] Yang Q. Wang X. Yin S. Zhao X. Tan J. Chen A. (2016). Improved emotional conflict control triggered by the processing priority of negative emotion. Sci. Rep. 6:24302. 10.1038/srep24302 27086908 PMC4834577

[B74] Yücel M. A. Lühmann A. von Scholkmann F. Gervain J. Dan I. Ayaz H.. (2021). Best practices for fNIRS publications. Neurophotonics 8, 012101–012101. 10.1117/1.NPh.8.1.012101 33442557 PMC7793571

[B75] Zhang Y. Liao Q. V. Bellamy R. K. (2020). “Effect of confidence and explanation on accuracy and trust calibration in AI-assisted decision making,” in Proceedings of the 2020 Conference on Fairness, Accountability, and Transparency (New York, NY), 295–305. 10.1145/3351095.3372852

